# Exact Probabilities and Confidence Limits for Binomial Samples: Applied to the Difference between Two Proportions

**DOI:** 10.1100/tsw.2010.75

**Published:** 2010-09-01

**Authors:** Lorentz Jäntschi, Sorana D. Bolboacă

**Affiliations:** ^1^ Department of Chemistry, Technical University of Cluj-Napoca, Romania; ^2^ Department of Medical Informatics and Biostatistics, “Iuliu Hatieganu” University of Medicine and Pharmacy Cluj-Napoca, Romania

**Keywords:** confidence interval, binomial sample, exact probabilities method (Exact.p), 2×2 contingency table, difference between two proportions

## Abstract

An exact probabilities method is proposed for computing the confidence limits of medical binomial parameters obtained based on the 2×2 contingency table. The developed algorithm was described and assessed for the difference between two binomial proportions (a bidimensional parameter). The behavior of the proposed method was analyzed and compared to four previously defined methods: Wald and Wilson, with and without continuity corrections. The exact probabilities method proved to be monotonic in computing the confidence limits. The experimental errors of the exact probabilities method applied to the difference between two proportions has never exceeded the imposed significance level of 5%.

### INTRODUCTION

The problem of computing confidence limits for a binomial success probability is not new; however, a consensus has not yet been reached. Most methods of computing the confidence interval are implemented in order to exclude both tails, but they could also be expressed to exclude only one tail. The confidence interval is used to report research results as a criterion of assessment of the trustworthiness or robustness of the finding[1,2], allowing a better interpretation of the results.

The following approaches are frequently used to construct the confidence interval for binomial proportions:

- Cumulative probabilities of a binomial distribution (Clopper and Pearson construct confidence limits by intersecting one-sided lower and upper intervals)[3]
- Approximation of binomial distribution with normal distribution (Wald method[4], also as normal approximation interval)

- Improvement of normal approximation interval (Wilson, the coverage probability is closer to the nominal value[5,6]; Agresti and Coull[7], Vollset[8], Bolboacă and Jäntschi[9], Hall[10], Gart and Nam[11])
- Exact intervals for binomial distribution (Sterne[12], Crow[13], Blyth and Still[14], Thomas[15], Korn and Graubard[16])
- Exact interval based on Poisson distribution (Crow and Gardner[17], Krishnamoorthy andLee[18])
- Optimized confidence interval (using two triangulations and simultaneously varying two pairs of three variables[19])
- Refinement methods (Casella[20], Casells and Robert[21], Newcombe[22])
- Asymptotic distribution (Sato[23], Cai[24])

By approximating binomial distributions with normal distributions, shorter intervals are obtained, but the coverage probability under the nominal level cannot be reduced. The Wald method, besides its inconveniences (poor coverage and poor accuracy), is widely used to report the confidence limits of medical parameters[25,26] implemented in various statistical programs. The main inconveniences of the Wald method used to compute confidence limits for binomial proportions are negative lower limits when the proportion is close to 1, upper limit higher than 1 when the proportion is close to 1[27,28], and a coverage from 65 to 85% instead of the desired 95% for small sample sizes ($n < 20$).

The bidimensional binomial distribution law is used in medical studies when the results obtained by medical research are compared with the results obtained by other similar research. In this case, the expression of the medical binomial parameter accompanied by the confidence interval must be evaluated. The difference between two proportions is commonly used to report results in medical studies. Wald and its corrected form are the most commonly used methods for confidence limits although they have poor coverage and accuracy. Therefore, the Newcombe-Wilson methods with and without continuity correction are recommended[29,30].

The problem of computing confidence limits on small samples for bidimensional binomial distribution is still investigated by many researchers. Troendle and Frank developed a conditionally exact tail method using both tails simultaneously[31]. The validity of various methods of constructing an approximate confidence interval for odds ratio, where hypergeometric sampling is applied, was discussed by Brown[32]. Lawson conducted an analysis on 10 exact and asymptotic distribution methods of computing the confidence interval for the odds ratio and concluded that Woolf's method[33], with the Haldane[34] and Anscombe[35] corrections, performed well especially as far as the length of the confidence interval was concerned[36]. The Laplace method[37] is recommended by Lewis and Sauro[38] when there is no expectation of the completion rate, by Wilson when the expectation rate is smaller than 0.5, and by Laplace when it is higher than 0.9. Otherwise, the maximum likelihood should be used[39]. Agresti[40] recommended using confidence methods for proportions, for the difference between proportions, and for odd ratios in three situations: when a lower bound on a coverage probability is desired, when an actual coverage probability near the nominal level is desired, and when teaching in a classroom. The confidence interval for the risk difference and attributable risk are frequently computed based on the Wald method[41], while recommendations are made according to sample size and value of the estimators[42].

The aim of our research was to develop an exact probabilities method (algorithm) in order to compute confidence limits for expressions of two binomial proportions. The algorithm was used to obtain the confidence limits for the difference between two proportions. It was also compared with the Wald and Wilson methods, with and without continuity corrections.

## MATERIAL AND METHODS

### Binomial and Bidimensional Binomial Distribution

A binomial sample was generated (*m* experiments are done and the number of *x* successes are counted). If *p* is the probability of success and *q* is the probability of failure (where $q = 1\text{-}p$) in the population, the probability to obtain *x* successes for a sample size of *m* is given by the formula presented in Eq. 1.

$$\text{Binomial}(\text{m},\text{p},\{\text{x}\}) = \frac{m!}{x!(m-x!)} p^{x}(1-p)^{m-x} \tag{1}$$

Using *x* as an unbiased estimator for the population mean ($m{\cdot}p$) and performing independent repetitions of a binomial experiment with the same sample size (*m*), the probability of *y* occurrence is given by the formula in Eq. 2.

$$\text{P}_\text{B}(\text{y},\text{x},\text{m}) = \frac{\text{m}!}{\text{y}!(\text{m}-\text{y})!}\left(\frac{\text{x}}{\text{m}}\right)^{\text{y}}\left(1-\frac{\text{x}}{\text{m}}\right)^{\text{m}-\text{y}} \tag{2}$$

A bidimensional binomial distribution could be obtained when the cases (noted x and y) are extracted from two samples of sizes m and n. The main hypothesis in medical studies is that the two samples (m and n) belong to the same population. The hypothesis that must be verified is: “Is there a significant difference between the two samples under the hypothesis that both are representative for the population?”

The probability that the binomial variable X (sample size of m and mean of x) and Y (sample size of n and mean of y) occur simultaneously and independently is given by Eq. 3.

$$\text{P}_\text{BB}(\text{X},\text{x},\text{m},\text{Y},\text{y},\text{n}) = \text{P}_\text{B}(\text{X},\text{x},\text{m})\cdot\text{P}_\text{B}(\text{Y},\text{y},\text{n}) \tag{3}$$

where $P_{BB}$ = probability of bibinomial distribution.

The probability that pairs (*X*,*Y*) occur in sample sizes of 10 ($m = n = 10$) was obtained by applying Eq. 3 (Fig. 1).

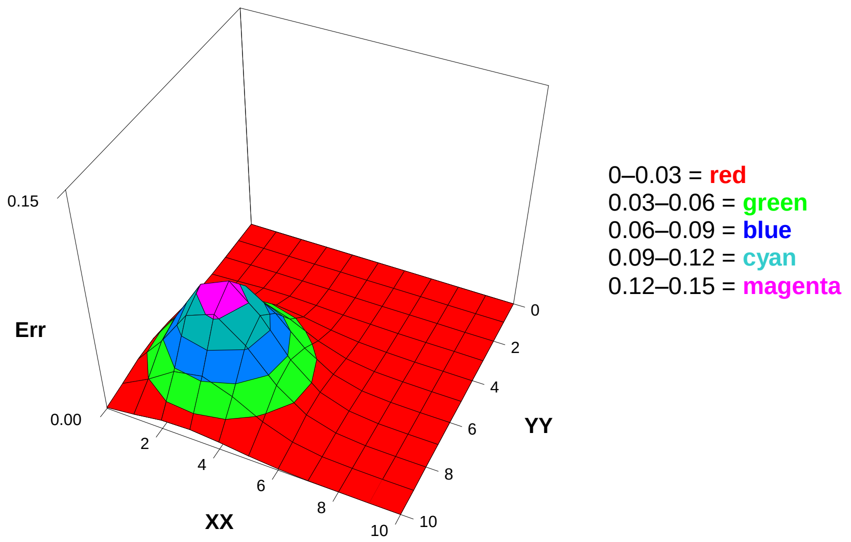

**FIGURE 1.** Bidimensional probability function; event: simultaneous extraction of *X* and *Y* from independent binomial samples (*x*,*10*) and (*y*,*10*). Probability for X = 3 and Y = 7.

### Algorithm of the Exact Probabilities Method

Confidence limits depend on the binomial distribution function calculated based on the values of the studied variables (as is shown in Fig. 1).

The lower and upper boundaries of the confidence interval could be obtained by the sum of the probabilities of simultaneous occurrence of values adjacent to the binomial values observed in the investigated samples (the sum of the segments on the plane XOY in Fig. 1).

A series of home-made PHP (recursive acronym for Hypertext Preprocessor) programs were developed in order to compute the confidence lower and upper limits at a significance level of 5%. The algorithm was developed and implemented after a previous analysis.

The algorithm is presented below:

```
for every observation vx from sample m
   for every observation vy from sample n
      for every random X drawn from the binomial distribution (vx,m)
         for every random Y drawn from the binomial distribution (vy,n)
            compute P_BB(vx,X,m,vy,Y,n) given by Eq. 3
            compute a medical-like indicator (MLI) using a formula for X,
         m, Y, n: MLI=MLI(X,m,Y,n)
      sort the table (ascending, maintaining associations) containing P_BB,
  and MLI by MLI values (procedure done in Table 3)
      group (summing the P_BB values) the values from sorted table by MLI
  values (procedure done in Table 4) - named TabSortSum (containing MLI and
  ΣP_BB)
      use (set) two keys: one for the top of the table (key is 1) and one
  for the bottom of the table (key is count of distinct MLI values), key_low
  & key_upp
      Set cumulated_probability to 0.0
      Repeat
         if entry key_low is lower than entry key_upp in Table TabSortSum
     for ΣP_BB (TabSortSum[key_low, ΣP_BB] < TabSortSum[key_upp, ΣP_BB]) then
            if cumulated_probability + TabSortSum[key_low, ΣP_BB] >
        desired_probability then break
            cumulated_probability += TabSortSum[key_low, ΣP_BB]
            key_low++
            continue
         if entry key_low is higher than entry key_upp in Table TabSortSum
     for ΣP_BB (TabSortSum[key_low, ΣP_BB] > TabSortSum[key_upp, ΣP_BB]) then
            if cumulated_probability + TabSortSum[key_upp, ΣP_BB] >
        desired_probability then break
            cumulated_probability += TabSortSum[key_upp, ΣP_BB]
            key_upp--
            continue
         if entry key_low is higher than entry key_upp in Table TabSortSum
     for ΣP_BB (TabSortSum[key_low, ΣP_BB] = TabSortSum[key_upp, ΣP_BB]) then
            if cumulated_probability + TabSortSum[key_low, ΣP_BB] >
        desired_probability then break
            cumulated_probability += TabSortSum[key_low, ΣP_BB]
            key_low++
            if cumulated_probability + TabSortSum[key_upp, ΣP_BB] >
        desired_probability then break
            cumulated_probability += TabSortSum[key_upp, ΣP_BB]
            key_upp--
            continue
      Until (false)
```

### Algorithm Assessment

The algorithm for computing the confidence interval was assessed on the excess risk (a measure of the association between a specified risk factor and a specified outcome)[43].

The excess risk is computed based on a 2×2 contingency table by using the formulas presented by Eq. 4.

$$ER = (bc-ad)/[(a+b)(c+d)] \quad (4)$$

where ER = excess risk; a = true positive; b = false positive; c = false negative; d = true negative.

The mathematical function associated to Eq. 4 is presented in Eq. 5.

$$f_4(X_1,X_2,X_3,X_4) = f_1(X_3,X_4)-f_1(X_1,X_2) \quad (5)$$

where $f_4$ = mathematical function for Eq. 4; $f_1(X_1,X_2) = X_1/(X_1+X_2)$, $f_1(X_3,X_4) = X_3/(X_3+X_4)$.

The substituted function associated to Eq. 4 and used by the proposed algorithm is presented in Eq. 6.

$$y/n-x/m \quad (6)$$

where $x$, $y$ = binomial distributed variables; $m$, $n$ = sizes of the samples.

Two experiments were conducted for equal sample sizes of 15 ($m = n = 15$):

1. ($0 \leq x \leq 15$,15,14,15), where $x$ varied from 0 to 15, $y$ had to be equal to 14, and $m$ and $n$ sample size were equal to 15
2. ($x_{random}$,15,$y_{random}$,15), where $x$ and $y$ were random variables and the sample sizes $m$ and $n$ were equal to 15

The following methods for computing the 95% confidence interval (significance level of 5%) were applied: exact probabilities (abbreviated as Exact.p) (proposed algorithm); Wald method (abbreviated as DWald)[4]; Wald with continuity correction (abbreviated as DWaldC; used only in the random experiment)[30]; Wilson (abbreviated as DWilson)[5]; and Wilson with continuity correction (abbreviated as DWilsonC)[29].

Four parameters were computed and assessed: the value of excess risk, the lower and upper 95% boundaries for each method ($\alpha$ = 5%), and the associated error for each method.

## RESULTS

### Examples of Bidimensional Binomial Distribution

The bidimensional probability matrix was constructed in order to meet the research objective. The bidimensional probability matrix obtained by applying Eq. 2, which corresponds to the extraction of the value ($Y$) from the binomial sample of ($y = 1$, $n = 10$) (see rows in the matrix) simultaneously with the extraction of the value ($X$) from the binomial sample of ($x = 9$, $m = 10$) (see columns in the matrix), is presented in Table 1. The intersection of a row with a column contains the values obtained by applying Eq. 3.

The probabilities associated with a bidimensional binomial distribution ($[x,m]$,$[y,n]$) could be used to calculate probabilities for any mathematical function of the $ff(x,m,y,m)$ type.

The values of probabilities for Eq. 6 when $m = 5$, $n = 5$, $x = 1$, $y = 2$ ($f4[1,5,2,5] = 2.00 \cdot 10^{-1}$) are included in Table 2.

The data in Table 2 are presented in ascending order according to the values of $f4(X,5,Y,5)$.

**TABLE 1**
**Bidimensional Probability Matrix (from Eq. 3)**

| **1.000** | **$3.49 \cdot 10^{-1}$** | **$3.87 \cdot 10^{-1}$** | **$1.94 \cdot 10^{-1}$** | **$5.74 \cdot 10^{-2}$** | **$1.12 \cdot 10^{-2}$** | **$1.49 \cdot 10^{-3}$** | **$1.38 \cdot 10^{-4}$** | **$8.75 \cdot 10^{-6}$** | **$3.65 \cdot 10^{-7}$** | **$9.00 \cdot 10^{-9}$** | **$1.00 \cdot 10^{-10}$** |
|---|---|---|---|---|---|---|---|---|---|---|---|
| **$1.00 \cdot 10^{-10}$** | $3.5 \cdot 10^{-11}$ | $3.9 \cdot 10^{-11}$ | $1.9 \cdot 10^{-11}$ | $5.7 \cdot 10^{-12}$ | $1.1 \cdot 10^{-12}$ | $1.5 \cdot 10^{-13}$ | $1.4 \cdot 10^{-14}$ | $8.7 \cdot 10^{-16}$ | $3.6 \cdot 10^{-17}$ | $9.0 \cdot 10^{-19}$ | $1.0 \cdot 10^{-20}$ |
| **$9.00 \cdot 10^{-9}$** | $3.1 \cdot 10^{-9}$ | $3.5 \cdot 10^{-9}$ | $1.7 \cdot 10^{-9}$ | $5.2 \cdot 10^{-10}$ | $1.0 \cdot 10^{-10}$ | $1.3 \cdot 10^{-11}$ | $1.2 \cdot 10^{-12}$ | $7.9 \cdot 10^{-14}$ | $3.3 \cdot 10^{-15}$ | $8.1 \cdot 10^{-17}$ | $9.0 \cdot 10^{-19}$ |
| **$3.65 \cdot 10^{-7}$** | $1.3 \cdot 10^{-7}$ | $1.4 \cdot 10^{-7}$ | $7.1 \cdot 10^{-8}$ | $2.1 \cdot 10^{-8}$ | $4.1 \cdot 10^{-9}$ | $5.4 \cdot 10^{-10}$ | $5.0 \cdot 10^{-11}$ | $3.2 \cdot 10^{-12}$ | $1.3 \cdot 10^{-13}$ | $3.3 \cdot 10^{-15}$ | $3.6 \cdot 10^{-17}$ |
| **$8.75 \cdot 10^{-6}$** | $3.1 \cdot 10^{-6}$ | $3.4 \cdot 10^{-6}$ | $1.7 \cdot 10^{-6}$ | $5.0 \cdot 10^{-7}$ | $9.8 \cdot 10^{-8}$ | $1.3 \cdot 10^{-8}$ | $1.2 \cdot 10^{-9}$ | $7.7 \cdot 10^{-11}$ | $3.2 \cdot 10^{-12}$ | $7.9 \cdot 10^{-14}$ | $8.7 \cdot 10^{-16}$ |
| **$1.38 \cdot 10^{-4}$** | $4.8 \cdot 10^{-5}$ | $5.3 \cdot 10^{-5}$ | $2.7 \cdot 10^{-5}$ | $7.9 \cdot 10^{-6}$ | $1.5 \cdot 10^{-6}$ | $2.1 \cdot 10^{-7}$ | $1.9 \cdot 10^{-8}$ | $1.2 \cdot 10^{-9}$ | $5.0 \cdot 10^{-11}$ | $1.2 \cdot 10^{-12}$ | $1.4 \cdot 10^{-14}$ |
| **$1.49 \cdot 10^{-3}$** | $5.2 \cdot 10^{-4}$ | $5.8 \cdot 10^{-4}$ | $2.9 \cdot 10^{-4}$ | $8.5 \cdot 10^{-5}$ | $1.7 \cdot 10^{-5}$ | $2.2 \cdot 10^{-6}$ | $2.1 \cdot 10^{-7}$ | $1.3 \cdot 10^{-8}$ | $5.4 \cdot 10^{-10}$ | $1.3 \cdot 10^{-11}$ | $1.5 \cdot 10^{-13}$ |
| **$1.12 \cdot 10^{-2}$** | $3.9 \cdot 10^{-3}$ | $4.3 \cdot 10^{-3}$ | $2.2 \cdot 10^{-3}$ | $6.4 \cdot 10^{-4}$ | $1.2 \cdot 10^{-4}$ | $1.7 \cdot 10^{-5}$ | $1.5 \cdot 10^{-6}$ | $9.8 \cdot 10^{-8}$ | $4.1 \cdot 10^{-9}$ | $1.0 \cdot 10^{-10}$ | $1.1 \cdot 10^{-12}$ |
| **$5.74 \cdot 10^{-2}$** | $2.0 \cdot 10^{-2}$ | $2.2 \cdot 10^{-2}$ | $1.1 \cdot 10^{-2}$ | $3.3 \cdot 10^{-3}$ | $6.4 \cdot 10^{-4}$ | $8.5 \cdot 10^{-5}$ | $7.9 \cdot 10^{-6}$ | $5.0 \cdot 10^{-7}$ | $2.1 \cdot 10^{-8}$ | $5.2 \cdot 10^{-10}$ | $5.7 \cdot 10^{-12}$ |
| **$1.94 \cdot 10^{-1}$** | $6.8 \cdot 10^{-2}$ | $7.5 \cdot 10^{-2}$ | $3.8 \cdot 10^{-2}$ | $1.1 \cdot 10^{-2}$ | $2.2 \cdot 10^{-3}$ | $2.9 \cdot 10^{-4}$ | $2.7 \cdot 10^{-5}$ | $1.7 \cdot 10^{-6}$ | $7.1 \cdot 10^{-8}$ | $1.7 \cdot 10^{-9}$ | $1.9 \cdot 10^{-11}$ |
| **$3.87 \cdot 10^{-1}$** | $1.4 \cdot 10^{-1}$ | $1.5 \cdot 10^{-1}$ | $7.5 \cdot 10^{-2}$ | $2.2 \cdot 10^{-2}$ | $4.3 \cdot 10^{-3}$ | $5.8 \cdot 10^{-4}$ | $5.3 \cdot 10^{-5}$ | $3.4 \cdot 10^{-6}$ | $1.4 \cdot 10^{-7}$ | $3.5 \cdot 10^{-9}$ | $3.9 \cdot 10^{-11}$ |
| **$3.49 \cdot 10^{-1}$** | $1.2 \cdot 10^{-1}$ | $1.4 \cdot 10^{-1}$ | $6.8 \cdot 10^{-2}$ | $2.0 \cdot 10^{-2}$ | $3.9 \cdot 10^{-3}$ | $5.2 \cdot 10^{-4}$ | $4.8 \cdot 10^{-5}$ | $3.1 \cdot 10^{-6}$ | $1.3 \cdot 10^{-7}$ | $3.1 \cdot 10^{-9}$ | $3.5 \cdot 10^{-11}$ |

**TABLE 2**
**Bidimensional Probability for Function *f4* for *m = 5*, *n = 5*, *x = 1*, *y = 2***

| X | Y | f4(X,5,Y,5) | $P_{BB}$(X,1,5,Y,2,5) | X | Y | f4(X,5,Y,5) | $P_{BB}$(X,1,5,Y,2,5) |
|---|---|---|---|---|---|---|---|
| 0 | 0 | $0.00 \cdot 10^{+0}$ | $2.55 \cdot 10^{-2}$ | 3 | 0 | $-6.00 \cdot 10^{-1}$ | $3.98 \cdot 10^{-3}$ |
| 0 | 1 | $2.00 \cdot 10^{-1}$ | $8.49 \cdot 10^{-2}$ | 3 | 1 | $-4.00 \cdot 10^{-1}$ | $1.33 \cdot 10^{-2}$ |
| 0 | 2 | $4.00 \cdot 10^{-1}$ | $1.13 \cdot 10^{-1}$ | 3 | 2 | $-2.00 \cdot 10^{-1}$ | $1.77 \cdot 10^{-2}$ |
| 0 | 3 | $6.00 \cdot 10^{-1}$ | $7.55 \cdot 10^{-2}$ | 3 | 3 | $0.00 \cdot 10^{+0}$ | $1.18 \cdot 10^{-2}$ |
| 0 | 4 | $8.00 \cdot 10^{-1}$ | $2.52 \cdot 10^{-2}$ | 3 | 4 | $2.00 \cdot 10^{-1}$ | $3.93 \cdot 10^{-3}$ |
| 0 | 5 | $1.00 \cdot 10^{+0}$ | $3.36 \cdot 10^{-3}$ | 3 | 5 | $4.00 \cdot 10^{-1}$ | $5.24 \cdot 10^{-4}$ |
| 1 | 0 | $-2.00 \cdot 10^{-1}$ | $3.19 \cdot 10^{-2}$ | 4 | 0 | $-8.00 \cdot 10^{-1}$ | $4.98 \cdot 10^{-4}$ |
| 1 | 1 | $0.00 \cdot 10^{+0}$ | $1.06 \cdot 10^{-1}$ | 4 | 1 | $-6.00 \cdot 10^{-1}$ | $1.66 \cdot 10^{-3}$ |
| 1 | 2 | $2.00 \cdot 10^{-1}$ | $1.42 \cdot 10^{-1}$ | 4 | 2 | $-4.00 \cdot 10^{-1}$ | $2.21 \cdot 10^{-3}$ |
| 1 | 3 | $4.00 \cdot 10^{-1}$ | $9.44 \cdot 10^{-2}$ | 4 | 3 | $-2.00 \cdot 10^{-1}$ | $1.48 \cdot 10^{-3}$ |
| 1 | 4 | $6.00 \cdot 10^{-1}$ | $3.15 \cdot 10^{-2}$ | 4 | 4 | $0.00 \cdot 10^{+0}$ | $4.92 \cdot 10^{-4}$ |
| 1 | 5 | $8.00 \cdot 10^{-1}$ | $4.19 \cdot 10^{-3}$ | 4 | 5 | $2.00 \cdot 10^{-1}$ | $6.55 \cdot 10^{-5}$ |
| 2 | 0 | $-4.00 \cdot 10^{-1}$ | $1.59 \cdot 10^{-2}$ | 5 | 0 | $-1.00 \cdot 10^{+0}$ | $2.49 \cdot 10^{-5}$ |
| 2 | 1 | $-2.00 \cdot 10^{-1}$ | $5.31 \cdot 10^{-2}$ | 5 | 1 | $-8.00 \cdot 10^{-1}$ | $8.29 \cdot 10^{-5}$ |
| 2 | 2 | $0.00 \cdot 10^{+0}$ | $7.08 \cdot 10^{-2}$ | 5 | 2 | $-6.00 \cdot 10^{-1}$ | $1.11 \cdot 10^{-4}$ |
| 2 | 3 | $2.00 \cdot 10^{-1}$ | $4.72 \cdot 10^{-2}$ | 5 | 3 | $-4.00 \cdot 10^{-1}$ | $7.37 \cdot 10^{-5}$ |
| 2 | 4 | $4.00 \cdot 10^{-1}$ | $1.57 \cdot 10^{-2}$ | 5 | 4 | $-2.00 \cdot 10^{-1}$ | $2.46 \cdot 10^{-5}$ |
| 2 | 5 | $6.00 \cdot 10^{-1}$ | $2.10 \cdot 10^{-3}$ | 5 | 5 | $0.00 \cdot 10^{+0}$ | $3.28 \cdot 10^{-6}$ |

The data presented in Table 3 were reorganized according to the sum of the probabilities corresponding to the identical values of the *f4(X,m,Y,n)* function (Table 4). The cumulated probabilities from extreme values towards the value of the *f4(x,m,y,n)* function were also included in Table 4 (last column).

**TABLE 3**
**Bidimensional Probability Function: Data Sorted Ascending by *f4(X,5,Y,5)***

| X | Y | f4(X,5,Y,5) | $P_{BB}$(X,1,5,Y,2,5) | X | Y | f4(X,5,Y,5) | $P_{BB}$(X,1,5,Y,2,5) |
|---|---|---|---|---|---|---|---|
| 5 | 0 | $-1.00\cdot10^{+0}$ | $2.49\cdot10^{-5}$ | 3 | 3 | $0.00\cdot10^{+0}$ | $1.18\cdot10^{-2}$ |
| 4 | 0 | $-8.00\cdot10^{-1}$ | $4.98\cdot10^{-4}$ | 4 | 4 | $0.00\cdot10^{+0}$ | $4.92\cdot10^{-4}$ |
| 5 | 1 | $-8.00\cdot10^{-1}$ | $8.29\cdot10^{-5}$ | 5 | 5 | $0.00\cdot10^{+0}$ | $3.28\cdot10^{-6}$ |
| 3 | 0 | $-6.00\cdot10^{-1}$ | $3.98\cdot10^{-3}$ | 0 | 1 | $2.00\cdot10^{-1}$ | $8.49\cdot10^{-2}$ |
| 4 | 1 | $-6.00\cdot10^{-1}$ | $1.66\cdot10^{-3}$ | 1 | 2 | $2.00\cdot10^{-1}$ | $1.42\cdot10^{-1}$ |
| 5 | 2 | $-6.00\cdot10^{-1}$ | $1.11\cdot10^{-4}$ | 2 | 3 | $2.00\cdot10^{-1}$ | $4.72\cdot10^{-2}$ |
| 2 | 0 | $-4.00\cdot10^{-1}$ | $1.59\cdot10^{-2}$ | 3 | 4 | $2.00\cdot10^{-1}$ | $3.93\cdot10^{-3}$ |
| 3 | 1 | $-4.00\cdot10^{-1}$ | $1.33\cdot10^{-2}$ | 4 | 5 | $2.00\cdot10^{-1}$ | $6.55\cdot10^{-5}$ |
| 4 | 2 | $-4.00\cdot10^{-1}$ | $2.21\cdot10^{-3}$ | 0 | 2 | $4.00\cdot10^{-1}$ | $1.13\cdot10^{-1}$ |
| 5 | 3 | $-4.00\cdot10^{-1}$ | $7.37\cdot10^{-5}$ | 1 | 3 | $4.00\cdot10^{-1}$ | $9.44\cdot10^{-2}$ |
| 1 | 0 | $-2.00\cdot10^{-1}$ | $3.19\cdot10^{-2}$ | 2 | 4 | $4.00\cdot10^{-1}$ | $1.57\cdot10^{-2}$ |
| 2 | 1 | $-2.00\cdot10^{-1}$ | $5.31\cdot10^{-2}$ | 3 | 5 | $4.00\cdot10^{-1}$ | $5.24\cdot10^{-4}$ |
| 3 | 2 | $-2.00\cdot10^{-1}$ | $1.77\cdot10^{-2}$ | 0 | 3 | $6.00\cdot10^{-1}$ | $7.55\cdot10^{-2}$ |
| 4 | 3 | $-2.00\cdot10^{-1}$ | $1.48\cdot10^{-3}$ | 1 | 4 | $6.00\cdot10^{-1}$ | $3.15\cdot10^{-2}$ |
| 5 | 4 | $-2.00\cdot10^{-1}$ | $2.46\cdot10^{-5}$ | 2 | 5 | $6.00\cdot10^{-1}$ | $2.10\cdot10^{-3}$ |
| 0 | 0 | $0.00\cdot10^{+0}$ | $2.55\cdot10^{-2}$ | 0 | 4 | $8.00\cdot10^{-1}$ | $2.52\cdot10^{-2}$ |
| 1 | 1 | $0.00\cdot10^{+0}$ | $1.06\cdot10^{-1}$ | 1 | 5 | $8.00\cdot10^{-1}$ | $4.19\cdot10^{-3}$ |
| 2 | 2 | $0.00\cdot10^{+0}$ | $7.08\cdot10^{-2}$ | 0 | 5 | $1.00\cdot10^{+0}$ | $3.36\cdot10^{-3}$ |

**TABLE 4**
**The Sum of Probabilities from Table 3**

| f4(1,5,2,5) | f4(X,5,Y,5) | $\Sigma P_{BB}$(X,1,5,Y,2,5) |
|---|---|---|
| $2.00\cdot10^{-1}$ | $-1.00\cdot10^{+0}$ | $2.49\cdot10^{-5}$ |
| $2.00\cdot10^{-1}$ | $-8.00\cdot10^{-1}$ | $5.81\cdot10^{-4}$ |
| $2.00\cdot10^{-1}$ | $-6.00\cdot10^{-1}$ | $5.75\cdot10^{-3}$ |
| $2.00\cdot10^{-1}$ | $-4.00\cdot10^{-1}$ | $3.15\cdot10^{-2}$ |
| $2.00\cdot10^{-1}$ | $-2.00\cdot10^{-1}$ | $1.04\cdot10^{-1}$ |
| $2.00\cdot10^{-1}$ | $0.00\cdot10^{+0}$ | $2.15\cdot10^{-1}$ |
| $2.00\cdot10^{-1}$ | $2.00\cdot10^{-1}$ | $2.78\cdot10^{-1}$ |
| $2.00\cdot10^{-1}$ | $4.00\cdot10^{-1}$ | $2.24\cdot10^{-1}$ |
| $2.00\cdot10^{-1}$ | $6.00\cdot10^{-1}$ | $1.09\cdot10^{-1}$ |
| $2.00\cdot10^{-1}$ | $8.00\cdot10^{-1}$ | $2.94\cdot10^{-2}$ |
| $2.00\cdot10^{-1}$ | $1.00\cdot10^{+0}$ | $3.36\cdot10^{-3}$ |

### Exact Probabilities Method: Experimental Errors

The experimental errors associated with the 95% confidence boundaries, when the implemented algorithm for excess risk ($m = n = 15$) was computed, are shown in Fig. 2. The values of errors varied from 0.00 to 4.95, with a mean of 3.56 and a standard deviation of 0.93.

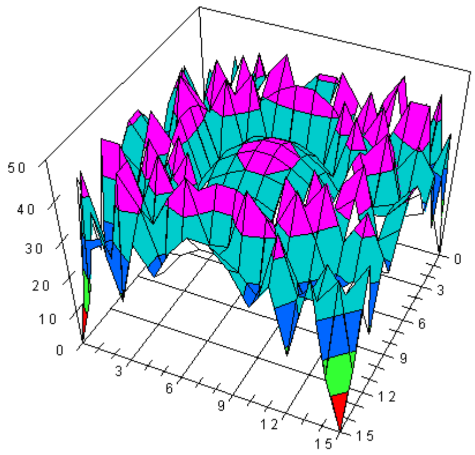

**FIGURE 2.** Distribution of experimental errors (in ‰): difference between two proportions *m = n = 15*.

### Results of the *(0≤x≤15,15,14,15)* Experiment

The 95% confidence boundaries for the *(0≤x≤15,15,14,15)* experiment were calculated and the results are presented in Fig. 3.

The following results were obtained when the confidence limits presented in Fig. 3 were analyzed to see whether they fit in the "golden standard" interval (the Exact.p method):

- The DWald method proved to be the closest method to the proposed Exact.p approach. The lower and upper limits obtained by applying the DWald method respected the Exact.p intervals for the lower and upper limit in 10 out of 16 cases (~63%).
- The Wilson methods (DWilson and DWilsonC) performed similarly with one exception (DWilson for *(13,15,14,15)*): the lower and upper boundaries were not included in the intervals obtained by the Exact.p method.

The descriptive statistics parameters associated with the experimental error for all the methods were computed and the results are presented in Table 5. The errors associated with the investigated methods (at a significance level of 5%) are presented in Fig. 4.

### Results of the *($x_{random}$,15,$y_{random}$,15)* Experiment

Forty-five values for *x* and *y* were assigned randomly by applying the (*0≤x≤m* and *0≤y≤n*) criteria. The obtained results are presented in Table 6. Table 6 also summarizes the inclusion of the limits obtained by each applied method into the range of boundaries obtained by the Exact.p method (under the assumption that the Exact.p method is the most restrictive method).

The variation of the experimental errors obtained by applying all the methods when computing the 95% confidence interval and when using the "|5-abs(Err)|" criterion is presented in Fig. 5.

### DISCUSSION

The aim of the research was to develop and assess an exact method for computing confidence boundaries for medical parameters computed on the 2×2 contingency table. The exact probabilities method proved to

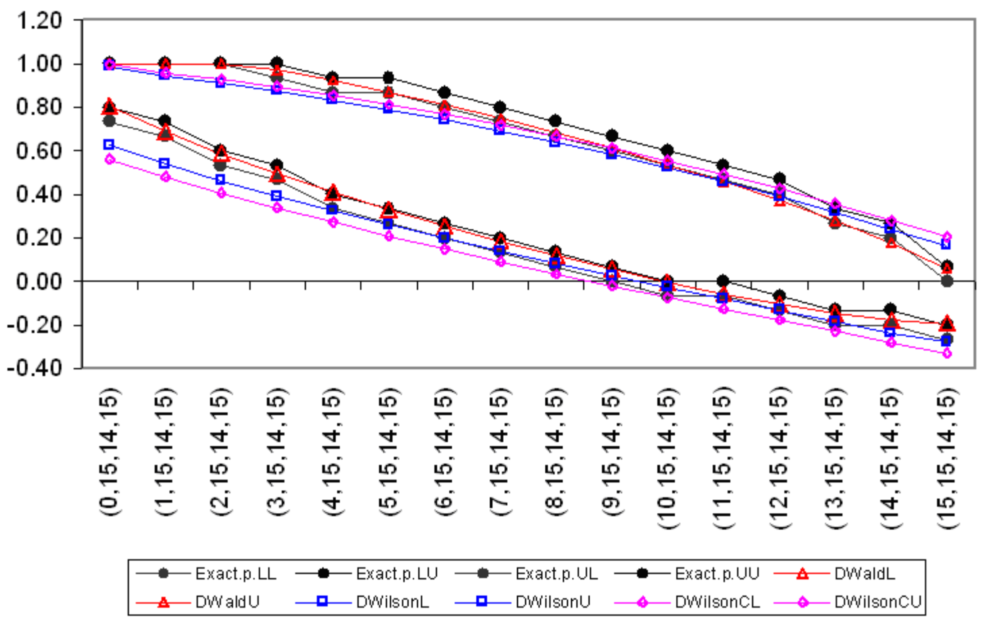

**FIGURE 3.** 95% Confidence boundaries for the *(0≤x≤15,15,14,15)* experiment.

**TABLE 5**
***(0≤x≤15,15,14,15)* Experiment: Summary of Experimental Error (expressed as %)**

| Method abb. | Min | Max | Mean | Standard Deviation |
|---|---|---|---|---|
| Exact.p | 1.49 | 4.80 | 3.27 | 0.99 |
| DWald | 2.50 | 7.38 | 4.65 | 1.86 |
| DWilson | 0.22 | 35.55 | 8.83 | 8.42 |
| DWilsonC | 0.18 | 35.53 | 6.82 | 9.18 |

be useful in computing the confidence limits on binomial samples with small sample sizes and provided reliable results. The Exact.p method provides confidence limits within 95% (significance level of 5%) or better no matter how small the sample size.

Computing the binomial probability matrix was the first step in applying the proposed method. The obtained probabilities were used later to construct confidence limits for binomial parameters. As expected, the repetitions of the value associated with the *f4(X,m,Y,n)* function used for exemplification (see Table 3) were not identified when the confidence limits were calculated. This observation applies to any function of the *ff* type (ff[x,m,y,m]).

The analysis of the results presented in Tables 1–3 revealed an important conclusion. The values of *X* and *Y* proved to be relevant only for the calculation of probabilities and for computing the values of functions. These values proved not to be important in the addition of probabilities used to construct the confidence limits. The adding of probabilities associated to bibinomial distribution values from the two extremes (lowest and highest - beginning and end of last column) proved to be a reliable solution in constructing the confidence limits by using the imposed probability and the experimental errors (see Table 4). These experimental errors are the real values of the probability errors obtained for the computed confidence limits. The experimental errors of the Exact.p method never exceeded the imposed significance level (4.95 was the maximum value, see Fig. 2).

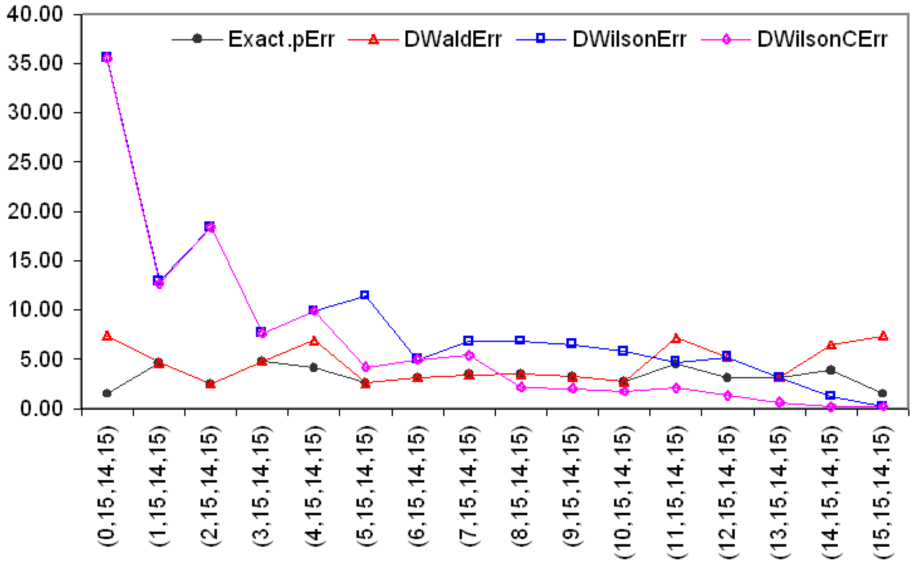

**FIGURE 4.** Distribution of errors associated to confidence limits computed with different methods.

The boundaries calculated by using the exact probabilities approach are, in fact, intervals for both the lower and the upper limit, as shown in Fig. 3. The Exact.p method is considered the "golden standard" in the evaluation of the 95% confidence interval according to the values of the experimental errors (Tables 5 and 6). The errors varied in the *(0≤x≤15,15,14,15)* experiment from 0.18% (DWilsonC) to 35.55% (DWilson), the lowest variation being obtained by the Exact.p method (3.32 was the difference between the maximum and the minimum error). The errors varied in the $(x_{random},15,y_{random},15)$ experiment from 0.03% (DWilsonC) to 35.55% (DWilson), the lowest variation was obtained by the Exact.p method (3.34 was the difference between the maximum and the minimum error).

The analysis of experimental errors (Tables 5 and 6, Figs. 4 and 5) led to the following observations:

- The experimental errors of the proposed method of constructing confidence limits has never exceeded the imposed significance (α = 5%).
- The DWald method proved to perform better than the DWilson method and its continuity correction, but similarly with the DWaldC method. The means of errors in the random experiment was 5.24 (1.86 standard deviation) when the DWald method was used and 5.00 (1.84 standard deviation) when the DWaldC method was used.

The DWald errors exceeded the imposed significance level of 5% in:

- Six out of 16 cases, with a maximum of 7.38 (for the *[0,15,14,15]*, *[0≤x≤15,15,14,15]* experiment)
- 27 out of 45 cases, with a maximum of 8.22 (for the *[13,15,12,15]*, $[x_{random},15,y_{random},15]$ experiment)

The errors exceeded the imposed significance level of 5% in:

- 25 out of 45 cases, with a maximum of 7.38 (for the *[14,15,12,15]*, $[x_{random},15,y_{random},15]$ experiment)

**TABLE 6**
**Experimental Errors and Coverage Obtained in the *($x_{random}$,15,$y_{random}$,15)* Experiment**

| x | m | y | n | y/n-x/m | Exact Probabilities Method | | Error | | | | | Coverage | | | |
|---|---|---|---|---|---|---|---|---|---|---|---|---|---|---|---|
| | | | | | LowerBound | UpperBound | Exact.p | DWald | DWaldC | DWilson | DWilsonC | DWald | DWaldC | DWilson | DWilsonC |
| 0 | 15 | 3 | 15 | 0.20 | $-6.67\cdot10^{-2},0.0\cdot10^{-1}$ | $4.00\cdot10^{-1},4.67\cdot10^{-1}$ | 1.81 | 1.81 | 1.81 | 1.81 | 0.42 | ✓ | ✓ | ✓ | × |
| 0 | 15 | 5 | 15 | 0.33 | $6.67\cdot10^{-2},1.33\cdot10^{-1}$ | $6.00\cdot10^{-1},6.67\cdot10^{-1}$ | 2.79 | 5.02 | 5.02 | 3.31 | 1.08 | × | × | × | × |
| 0 | 15 | 7 | 15 | 0.47 | $2.00\cdot10^{-1},2.67\cdot10^{-1}$ | $7.33\cdot10^{-1},8.00\cdot10^{-1}$ | 4.11 | 6.64 | 6.64 | 4.19 | 3.54 | × | × | × | × |
| 1 | 15 | 0 | 15 | –0.07 | $-2.67\cdot10^{-1},-2.00\cdot10^{-1}$ | $0.00\cdot10^{-1},6.67\cdot10^{-2}$ | 1.49 | 7.38 | 7.38 | 0.22 | 0.22 | × | × | × | × |
| 1 | 15 | 3 | 15 | 0.13 | $-1.33\cdot10^{-1},-6.67\cdot10^{-2}$ | $4.00\cdot10^{-1},4.67\cdot10^{-1}$ | 3.13 | 5.25 | 5.25 | 5.25 | 1.33 | × | × | × | × |
| 1 | 15 | 4 | 15 | 0.20 | $-6.67\cdot10^{-2},0.00\cdot10^{-1}$ | $4.67\cdot10^{-1},5.33\cdot10^{-1}$ | 4.52 | 7.18 | 7.18 | 4.73 | 2.07 | × | × | × | × |
| 1 | 15 | 15 | 15 | 0.93 | $7.33\cdot10^{-1},8.00\cdot10^{-1}$ | $\leq 1.00\cdot10^{0}$ | 1.49 | 7.38 | 7.38 | 35.55 | 35.53 | × | × | × | × |
| 2 | 15 | 2 | 15 | 0.00 | $-2.67\cdot10^{-1},-2.00\cdot10^{-1}$ | $2.67\cdot10^{-1},3.33\cdot10^{-1}$ | 3.68 | 5.80 | 5.80 | 5.80 | 1.56 | × | × | × | × |
| 2 | 15 | 7 | 15 | 0.33 | $-6.67\cdot10^{-2},0.00\cdot10^{-1}$ | $6.00\cdot10^{-1},6.67\cdot10^{-1}$ | 3.37 | 5.19 | 5.19 | 9.21 | 3.37 | × | × | × | ✓ |
| 2 | 15 | 12 | 15 | 0.67 | $3.33\cdot10^{-1},4.00\cdot10^{-1}$ | $8.67\cdot10^{-1},9.33\cdot10^{-1}$ | 4.83 | 7.89 | 2.34 | 10.84 | 10.38 | × | × | × | × |
| 2 | 15 | 14 | 15 | 0.80 | $5.33\cdot10^{-1},6.00\cdot10^{-1}$ | $\leq 1.00\cdot10^{0}$ | 2.50 | 2.50 | 2.50 | 18.37 | 18.37 | ✓ | ✓ | × | × |
| 3 | 15 | 15 | 15 | 0.80 | $5.33\cdot10^{-1},6.00\cdot10^{-1}$ | $\leq 1.00\cdot10^{0}$ | 1.81 | 1.81 | 1.81 | 17.14 | 3.60 | ✓ | ✓ | × | × |
| 4 | 15 | 1 | 15 | –0.20 | $-5.33\cdot10^{-1},-4.67\cdot10^{-1}$ | $0.00\cdot10^{-1},6.67\cdot10^{-2}$ | 4.52 | 7.18 | 7.18 | 4.52 | 2.07 | × | × | ✓ | × |
| 4 | 15 | 6 | 15 | 0.13 | $-2.67\cdot10^{-1},-2.00\cdot10^{-1}$ | $4.67\cdot10^{-1},5.33\cdot10^{-1}$ | 2.97 | 2.97 | 2.97 | 7.63 | 5.28 | ✓ | ✓ | × | × |
| 4 | 15 | 12 | 15 | 0.53 | $2.00\cdot10^{-1},2.67\cdot10^{-1}$ | $8.00\cdot10^{-1},8.67\cdot10^{-1}$ | 4.78 | 4.78 | 4.78 | 6.83 | 6.05 | ✓ | ✓ | × | × |
| 5 | 15 | 4 | 15 | –0.07 | $-4.00\cdot10^{-1},-3.33\cdot10^{-1}$ | $2.67\cdot10^{-1},3.33\cdot10^{-1}$ | 4.82 | 7.02 | 7.02 | 7.02 | 4.85 | × | × | × | × |
| 6 | 15 | 4 | 15 | –0.13 | $-5.33\cdot10^{-1},-4.67\cdot10^{-1}$ | $2.00\cdot10^{-1},2.67\cdot10^{-1}$ | 2.97 | 2.97 | 2.97 | 7.63 | 5.32 | ✓ | ✓ | × | × |
| 6 | 15 | 7 | 15 | 0.07 | $-3.33\cdot10^{-1},-2.67\cdot10^{-1}$ | $4.00\cdot10^{-1},4.67\cdot10^{-1}$ | 4.04 | 4.04 | 4.04 | 9.49 | 4.04 | ✓ | ✓ | × | ✓ |
| 6 | 15 | 10 | 15 | 0.27 | $-1.33\cdot10^{-1},-6.67\cdot10^{-2}$ | $6.00\cdot10^{-1},6.67\cdot10^{-1}$ | 3.47 | 3.47 | 3.47 | 5.95 | 5.95 | ✓ | ✓ | × | × |
| 7 | 15 | 3 | 15 | –0.27 | $-6.00\cdot10^{-1},-5.33\cdot10^{-1}$ | $6.67\cdot10^{-2},1.33\cdot10^{-1}$ | 4.52 | 6.70 | 6.70 | 9.02 | 4.64 | × | × | × | × |
| 7 | 15 | 15 | 15 | 0.53 | $2.00\cdot10^{-1},2.67\cdot10^{-1}$ | $7.33\cdot10^{-1},8.00\cdot10^{-1}$ | 4.11 | 6.64 | 6.64 | 4.11 | 3.38 | × | × | ✓ | × |
| 8 | 15 | 5 | 15 | –0.20 | $-6.00\cdot10^{-1},-5.33\cdot10^{-1}$ | $1.33\cdot10^{-1},2.00\cdot10^{-1}$ | 3.66 | 3.66 | 3.66 | 6.29 | 6.29 | ✓ | ✓ | × | × |
| 8 | 15 | 9 | 15 | 0.07 | $-3.33\cdot10^{-1},-2.67\cdot10^{-1}$ | $4.00\cdot10^{-1},4.67\cdot10^{-1}$ | 4.04 | 4.04 | 4.04 | 9.49 | 4.04 | ✓ | ✓ | × | ✓ |
| 8 | 15 | 12 | 15 | 0.27 | $-1.33\cdot10^{-1},-6.67\cdot10^{-2}$ | $5.33\cdot10^{-1},6.00\cdot10^{-1}$ | 4.52 | 6.70 | 6.70 | 8.99 | 4.52 | × | × | × | ✓ |
| 9 | 15 | 10 | 15 | 0.07 | $-3.33\cdot10^{-1},-2.67\cdot10^{-1}$ | $4.00\cdot10^{-1},4.67\cdot10^{-1}$ | 3.50 | 3.50 | 3.50 | 8.56 | 3.50 | ✓ | ✓ | × | ✓ |
| 10 | 15 | 1 | 15 | –0.60 | $-9.33\cdot10^{-1},-8.67\cdot10^{-1}$ | $-3.33\cdot10^{-1},-2.67\cdot10^{-1}$ | 2.62 | 2.62 | 2.62 | 11.58 | 5.05 | ✓ | ✓ | × | × |
| 10 | 15 | 13 | 15 | 0.20 | $-1.33\cdot10^{-1},-6.67\cdot10^{-2}$ | $4.67\cdot10^{-1},5.33\cdot10^{-1}$ | 4.36 | 4.36 | 4.36 | 4.36 | 2.85 | ✓ | ✓ | ✓ | × |
| 11 | 15 | 1 | 15 | –0.67 | $-9.33\cdot10^{-1},-8.67\cdot10^{-1}$ | $-4.00\cdot10^{-1},-3.33\cdot10^{-1}$ | 4.12 | 6.93 | 6.93 | 10.52 | 10.52 | × | × | × | × |
| 11 | 15 | 5 | 15 | –0.40 | $-7.33\cdot10^{-1},-6.67\cdot10^{-1}$ | $-6.67\cdot10^{-2},0.00\cdot10^{-1}$ | 4.65 | 6.95 | 6.95 | 9.31 | 4.88 | × | × | × | × |
| 11 | 15 | 8 | 15 | –0.20 | $-6.00\cdot10^{-1},-5.33\cdot10^{-1}$ | $1.33\cdot10^{-1},2.00\cdot10^{-1}$ | 3.13 | 3.13 | 3.13 | 5.57 | 5.57 | ✓ | ✓ | × | × |
| 11 | 15 | 14 | 15 | 0.20 | $-6.67\cdot10^{-2},0.00\cdot10^{-1}$ | $4.67\cdot10^{-1},5.33\cdot10^{-1}$ | 4.52 | 7.18 | 7.18 | 4.73 | 2.07 | × | × | × | × |
| 12 | 15 | 0 | 15 | –0.80 | $\geq-1.00\cdot10^{0}$ | $-6.00\cdot10^{-1},-5.33\cdot10^{-1}$ | 1.81 | 1.81 | 1.81 | 16.42 | 6.11 | ✓ | ✓ | × | × |
| 12 | 15 | 3 | 15 | –0.60 | $-9.33\cdot10^{-1},-8.67\cdot10^{-1}$ | $-3.33\cdot10^{-1},-2.67\cdot10^{-1}$ | 3.61 | 3.61 | 3.61 | 12.99 | 6.11 | ✓ | ✓ | × | × |
| 12 | 15 | 5 | 15 | –0.47 | $-8.00\cdot10^{-1},-7.33\cdot10^{-1}$ | $-1.33\cdot10^{-1},-6.67\cdot10^{-2}$ | 3.66 | 5.71 | 5.71 | 8.14 | 7.65 | × | × | × | × |
| 12 | 15 | 8 | 15 | –0.27 | $-6.00\cdot10^{-1},-5.33\cdot10^{-1}$ | $6.67\cdot10^{-2},1.33\cdot10^{-1}$ | 4.52 | 6.70 | 6.70 | 9.02 | 4.64 | × | × | × | × |
| 12 | 15 | 10 | 15 | –0.13 | $-4.67\cdot10^{-1},-4.00\cdot10^{-1}$ | $2.00\cdot10^{-1},2.67\cdot10^{-1}$ | 3.90 | 5.82 | 5.82 | 5.82 | 3.94 | × | × | × | × |
| 13 | 15 | 10 | 15 | –0.20 | $-5.33\cdot10^{-1},-4.67\cdot10^{-1}$ | $6.67\cdot10^{-2},1.33\cdot10^{-1}$ | 4.36 | 4.36 | 4.36 | 4.36 | 2.86 | ✓ | ✓ | ✓ | × |
| 13 | 15 | 12 | 15 | –0.07 | $-4.00\cdot10^{-1},-3.33\cdot10^{-1}$ | $2.00\cdot10^{-1},2.67\cdot10^{-1}$ | 2.59 | 8.22 | 2.59 | 2.59 | 2.59 | × | ✓ | ✓ | ✓ |
| 14 | 15 | 15 | 15 | 0.07 | $-6.67\cdot10^{-2},0.00\cdot10^{-1}$ | $2.00\cdot10^{-1},2.67\cdot10^{-1}$ | 1.49 | 7.38 | 7.38 | 0.22 | 0.03 | × | × | × | × |
| 15 | 15 | 4 | 15 | –0.73 | $-1.00\cdot10^{0},-9.33\cdot10^{-1}$ | $-5.33\cdot10^{-1},-4.67\cdot10^{-1}$ | 3.50 | 3.50 | 3.50 | 7.71 | 7.71 | ✓ | ✓ | × | × |
| 15 | 15 | 5 | 15 | –0.67 | $-9.33\cdot10^{-1},-8.67\cdot10^{-1}$ | $-4.00\cdot10^{-1},-3.33\cdot10^{-1}$ | 2.79 | 5.02 | 5.02 | 9.05 | 3.08 | × | × | × | × |
| 15 | 15 | 6 | 15 | –0.60 | $-8.67\cdot10^{-1},-8.00\cdot10^{-1}$ | $-3.33\cdot10^{-1},-2.67\cdot10^{-1}$ | 3.65 | 6.10 | 6.10 | 3.90 | 3.43 | × | × | × | × |
| 15 | 15 | 8 | 15 | –0.47 | $-8.00\cdot10^{-1},-7.33\cdot10^{-1}$ | $-2.67\cdot10^{-1},-2.00\cdot10^{-1}$ | 4.11 | 6.64 | 6.64 | 4.11 | 3.38 | × | × | ✓ | × |
| 15 | 15 | 10 | 15 | –0.33 | $-6.67\cdot10^{-1},-6.00\cdot10^{-1}$ | $-1.33\cdot10^{-1},-6.67\cdot10^{-2}$ | 2.79 | 5.02 | 5.02 | 2.79 | 1.08 | × | × | ✓ | × |
| 15 | 15 | 14 | 15 | –0.07 | $-2.67\cdot10^{-1},-2.00\cdot10^{-1}$ | $0.00\cdot10^{-1},6.67\cdot10^{-2}$ | 1.49 | 7.38 | 7.38 | 0.22 | 0.22 | × | × | × | × |

The DWald and DWaldC methods proved to obtain the highest values of the experimental errors (*[13,15,12,15]* and *[14,15,12,15]*, respectively). In these cases, the Exact.p method obtained experimental errors equal to 2.59 and 1.49%.

- The DWilson and DWilsonC methods had similar performances: the minimum value of the experimental errors was <1 while the maximum value of experimental error was >30. The DWilsonC method performed better in terms of exceeding the imposed significance level compared to DWilson, DWald, and DWaldC, as it exceeded the imposed significance level in only

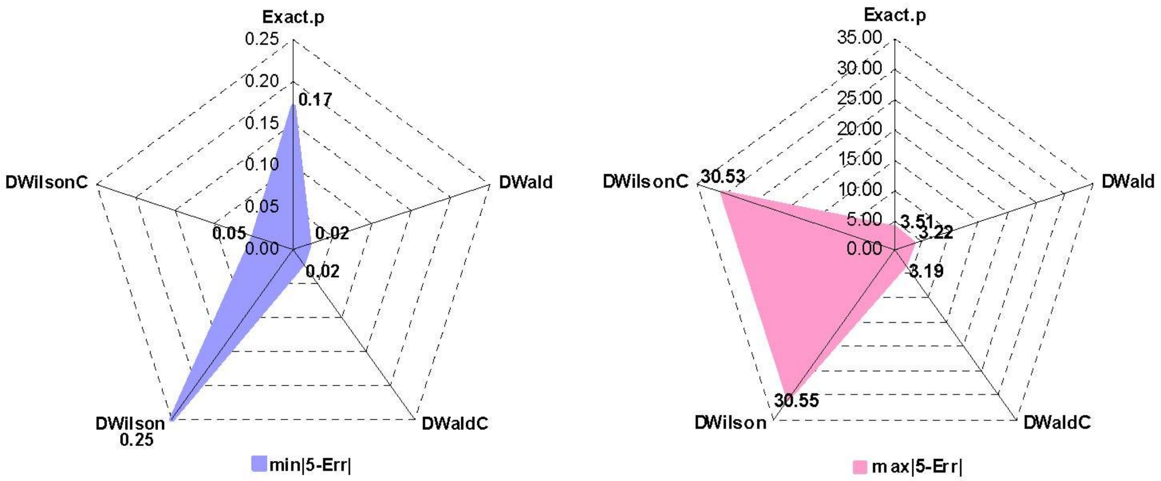

**FIGURE 5**. 5% Deviation of the experimental errors from the imposed significance level.

15 out of 45 cases in the random experiment. Furthermore, the mean of experimental errors in the random experiment was equal to 5, with a standard deviation of 5.65. The highest value of errors was obtained by both Wilson methods for *(1,15,15,15)* in the random experiment.

- The hierarchy of experimental errors for values ≤5% was Exact.p (100%), DWilsonC (67%), DWaldC (44%), DWald (40%), DWilson (36%).
- The classification in descending order, based on the maximum value of the errors associated with the 95% confidence limits, was: DWilson (35.55 for *[1,15,15,15]*), DWilsonC (35.53 for *[1,15,15,15]*), DWald (8.22 for *[13,15,12,15]*), DwaldC (7.38 for *[1,15,0,15]*), Exact.p (4.83 for *[2,15,12,15]*).

The confidence limits obtained in the random experiment were checked to see whether they fit in the “golden standard” interval (the Exact.p method, according to the values of the experimental errors). The following proved to be true (see Table 6):

- The DWaldC method had the highest inclusion: 19 out of 45 cases. It was closely followed by the DWald method: 18 out of 45 cases.
- The DWilson method performed slightly better than DWilsonC: eight inclusions out of 45 compared with six inclusions out of 45.

The newly introduced method for constructing the confidence limits for discrete distributions using the probability distribution matrix proved to respect, without any exception, the imposed significance level. The proposed algorithm was assessed in two experiments by using the excess risk parameter, which is a binomial parameter computed on the 2×2 contingency table. The algorithm was assessed in terms of the difference between two proportions, but it could also be applied to construct the confidence limits of any parameter computed based on the 2×2 contingency table, since computing the confidence limits is based on the probability matrix.

### CONCLUSIONS

The proposed algorithm obtained the exact domain for the lower and upper limits of the confidence interval for $f$, $X$, $Y$, $m$, $n$, $\alpha$, and their associated experimental errors. The implemented algorithm required that the desired value for the experimental errors was as close as possible to the imposed significance level α without exceeding this level.

The proposed algorithm proved to be monotone and never exceeded the imposed significance level in small sample sizes (*m = n = 10, m = n = 15*). Further studies will be conducted in order to analyze the behavior of the implemented algorithm on different sample sizes and different discretely distributed parameters.
